# Outcome of Laparoscopy in Infertile Couples attending a Teaching Hospital in Eastern Nepal: A Descriptive Cross-sectional Study

**DOI:** 10.31729/jnma.5542

**Published:** 2020-11-30

**Authors:** Shanti Sunuwar Subedi, Rakina Bhansakarya, Prajmi Shrestha, Sajjan Kumar Sharma

**Affiliations:** 1 Department of Obstetrics and Gynecology, Nobel Medical College Teaching Hospital, Biratnagar, Nepal

**Keywords:** *cyst*, *endometriosis*, *female*, *laparoscopy*, *pregnancy*

## Abstract

**Introduction:**

Infertility is a global health issue and a socially destabilizing condition for couples with several stigmas including medical, social, psychological burdens and a marital disharmony. The aim was to study the outcome of laparoscopy in infertile females attending Nobel Medical College as laparoscopy is considered as a gold standard in investigation and treatment of infertility.

**Methods:**

A descriptive cross-sectional study was carried out in the department of Obstetrics and Gynaecology over a period of May 2018-April 2020, where the outcomes of laparoscopy in infertile females were studied. All the patients with abnormal HSG, unexplained infertility and adnexal mass in the background of infertility were enrolled.

**Results:**

Of the 100 infertile patients who underwent laparoscopy, 62 (62%) had evidence of tubal disease as documented by unilateral or bilateral block, 63 (63%) had peritubal adhesions and hydrosalpinx in 15 (15%). Associated pelvic pathology like endometriosis in 50 (50%) in the form of cyst, adhesions and complete and partial obliteration of Pouch of Douglas.

**Conclusions:**

Laparoscopy is definitely an effective diagnostic tool of tubal and pelvic pathology. Laparoscopy is recommended for all infertile females with suspected tubal factor and moreover it provides opportunity to correct the condition in possible cases.

## INTRODUCTION

Infertility is one of the major health problems worldwide, including Nepal. The average prevalence of infertility in developing countries is estimated to be around 6.99.3%.^1^ Approximately 15% of couples attempting their first pregnancy meet with failure. A global review of infertility from the World Fertility Survey estimated infertility as 4% in Bangladesh, 6% in Nepal, 5% in Pakistan and 4% in Sri Lanka.^2^

Infertility is defined as failure to achieve pregnancy after a year of unprotected intercourse. This is further classified as primary if the couples fail to achieve previous pregnancy and secondary if a prior pregnancy, although not necessarily a live birth, has occurred.^3^ Leading causes of infertility include tubal disease, ovulatory disorders, uterine or cervical factors, endometriosis and male factor infertility.^4,5,6^ Laparoscopy is an essential step and a standard procedure in the investigation and evaluation of infertile females before initiating infertility problems.^7,8^ According to World Health Organization (WHO) guidelines, diagnostic laparoscopy is still recommended as minimal requirement in the investigation of female infertility.^9^ In the case of unexplained infertility, laparoscopy offers an excellent modality through direct visualization to rule out any hidden pathology.

This study aims the role of laparoscopy in female infertility.

## METHODS

A descriptive cross-sectional study was carried out in the department of Obstetrics and Gynecology, Nobel Medical College Teaching Hospital, Biratnagar from May 2018- April 2020. Ethical clearance was taken from the hospital IRC with Reference Number 142/2018. After obtaining informed consent, all the patients were prepared for the procedure during the proliferative phase of the menstrual cycle. All the patients with the history of failure to conceive for a period of one year were included in the study. The sample size was calculated as per the following formula:

Convenience sampling was done, and the sample size (n) was calculated as,

$$\begin{array}{ll} \text{n} & \text{= }{\text{Z}}^{\text{2}}\times \text{p}\times \text{q}/{\text{e}}^{\text{2}}\\ & \text{= }{\left(1.64\right)}^{\text{2}}\times 0.5\times 0.5/{(\text{0.1)}}^{\text{2}}\\ & \text{= }67 \end{array}$$

Where,

Z = 1.64 at 90% Confidence Interval
p = prevalence 50%
q = 1-p
e = margin of error, 10%

The sample size came as sixty-seven (n = 67). The detail history of the couples and physical examination of the female partner was done and for male partner, examination was done by urologist if at all semen analysis was found to be abnormal. All the patients had undergone semen analysis after three days of abstinence, abdominal ultrasound, hysterosalpinography on day 6-day 11 and hormonal assessment included thyroid function test and serum prolactin as a routine. AMH done in selected patients depending on the age. Diagnostic laparoscopy with chromopertubation done if HSG was abnormal and in case of unexplained infertility and if adnexal mass was noted in clinical and ultrasound examination.

The relevant clinical findings were documented and finally data were extracted from the same and analyzed using SPSS version 26. The descriptive statistical analysis was done. Frequency and percentages were calculated for binary data whereas mean and standard deviation were calculated for continuous data.

## RESULTS

A total of one hundred infertile females with abnormal HSG, unexplained infertility and if adnexal mass noted on clinical and ultrasound examination in the background of infertility were included in the study.

**Figure 1:**
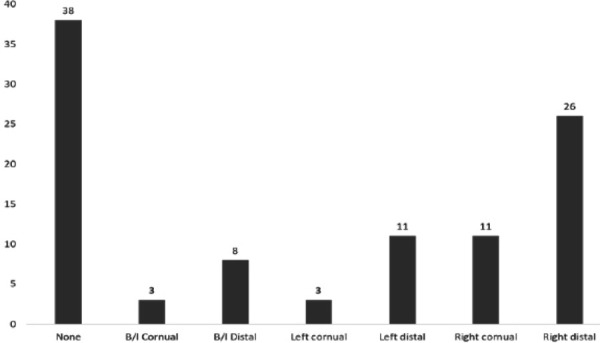
Different sites of block.

**Figure 2:**
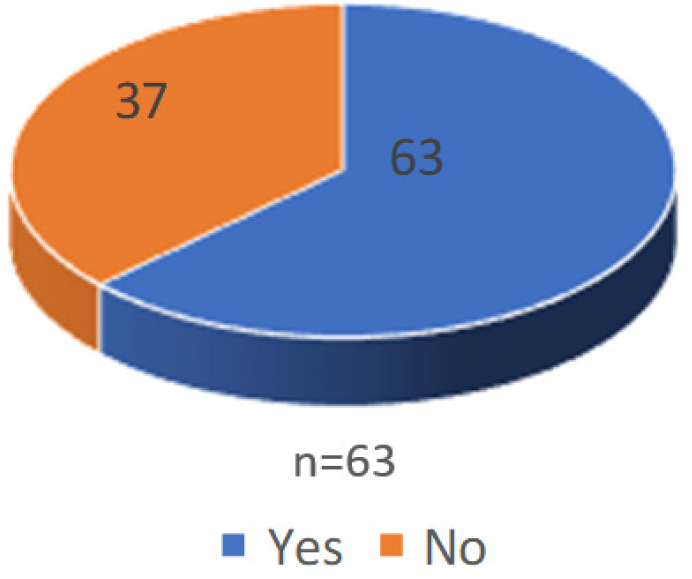
Percentage of peritubal adhesion.

**Figure 3:**
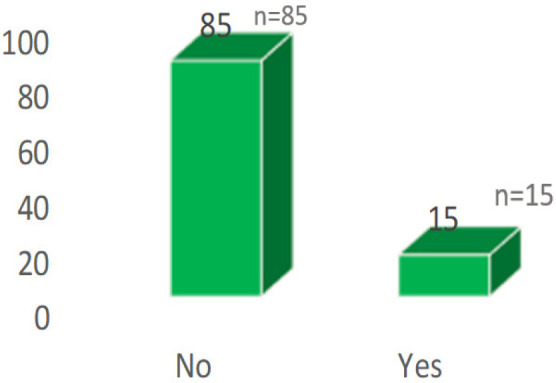
Percentage of Hydrosalpinx.

Of the patients with tubal factor, 62 (62%) had block in one or both tubes, right distal tubal block was the commonest finding. 63 (63%) had peritubal adhesions and hydrosalpinx was observed in 15 (15%) (Figure 1, 2 and 3).

Endometriosis was observed in 50 (50%) of them underwent laparoscopy in the form of endometriotic cyst, adhesions and partial and complete obliteration of the pouch of Douglas.

Associated lesions diagnosed during evaluation were dermoid cyst in 25 (25%) and myoma in 13 (13%).

**Figure 4:**
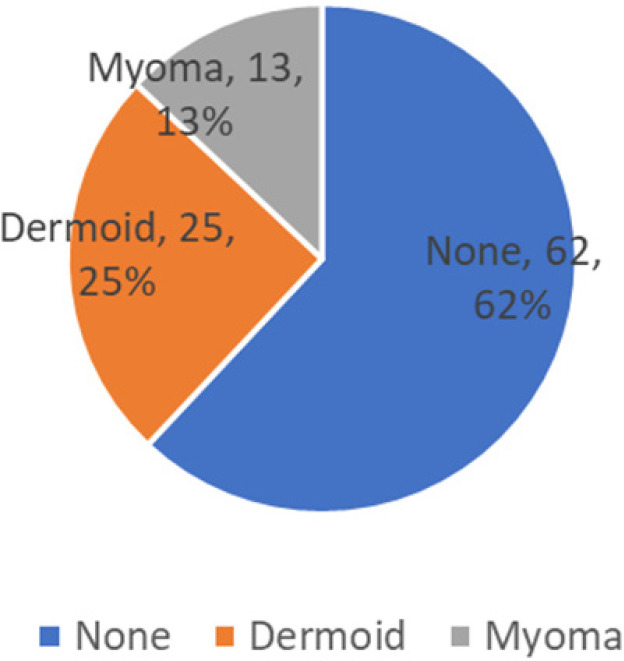
Associated lesions diagnosed by laparoscopy.

The mean age of the patients was 28.61 years (range from 16-47 years). Primary infertility was more 51 (51%) than secondary infertility 49 (49%) (Table 1).

**Table 1:** Age group and type of infertility (n=100).

Variables	Frequency
Age ( years)	
< 20	14
20-29	45
30-39	31
>40	10
Type of infertility:	
Primary	51
Secondary	49

Surprisingly 54 (54%) with infertility females were housewives by occupation as compared to working females and no obvious association were found for the same. Mean age of infertility was found be 28.61 years (Table 2).

**Table 2:** Socio-Demographic Characteristics.

Mean age of infertility	28.61 years
Minimum age	16 years
Maximum age	47 years
Mean duration of infertility	5.14 years

## DISCUSSION

Advances in imaging modalities have enabled accurate diagnosis of uterine and adnexal disease, thus redefining the role of laparoscopy. These techniques include Computed tomography, Magnetic resonance imaging. Hysterosalpingography, Hysterosalpingo-contrast sonography and two, three dimensional ultrasound are inexpensive, fast and well tolerated methods of determining tubal patency, though their value when compared to laparoscopy is still a matter of debate.^10^

It is generally accepted that diagnostic laparoscopy is the gold standard in diagnosing tubal pathology and other intra-abdominal causes of infertility.^11,12,13^

In this study of the 100 infertile females who underwent laparoscopy, 62% had tubal block in one or both tubes, 63% had peritubal adhesions and hydrosalpinx was noted in 15%. These observations illustrate that the prevalence of tubal pathology in women complaining of infertility is high in our community. The incidence is similar to the studies done by Shetty et al,^14^ Choudhari et al.^15^ Right distal tubal block was found in 41.93% and bilateral block in 17.74% cases in this study which is different from the study done by Foroozanfard et al^16^ where bilateral tubal patency was observed in 72.6% and unilateral and bilateral patency in 27.40%. In a similar study done by M Heis et al^17^ found that 6.04% had bilateral tubal occlusion and 5.69% had unilateral occlusion.

Peritubal adhesion was observed in 63% of cases in this study. Peritubal adhesions are usually formed as a result of inflammation from pelvic inflammatory disease or surgery. These adhesions disturb the anatomic relationship between the tubes and ovaries culminating in infertility. Pelvic adhesions around the ovaries may interrupt the blood supply to the ovaries, thereby resulting in impaired gonadotropin access to target organs (Nagata et al. 1998).^18^ In addition, adhesions may lead to impaired access to the ovary during oocyte pick-up, which results in a decreased number of collected oocytes (Daniell et al. 1983).^19^

Hydrosalpinges adversely affect reproductive outcomes and several factors have been implicated, including a mechanical interference with implantation and toxic effects on the endometrium and embryo. (Beyler et al^20^. 1997; Meyer et al. 1997^21^). This study observed the same in 15% of cases. The negative effects of hydrosalpinges on spontaneous conceptions and IVF pregnancies are well established (Beyler et al.^20^ 1997; Meyer et al.^21^ 1997).

Endometriosis was observed in 50% of cases in the form of endometriotic cyst, adhesions with partial and complete obliteration of pouch of Douglas. Endometriosis affects 2.5-3.3 percent of women of reproductive age^22^ and is diagnosed in 20-68 percent of the women studied for infertility.^23^

Additional findings obtained during laparoscopy in the study were desmoids in 25% and myoma in 13 % of the cases.

In our study of 100 patients, 51% were of primary and 49% of secondary infertility which were comparable with the studies done by Foroozanfard et al^16^, M heiss et al,^17^ Gokhan Goynumer et al.^24^

## CONCLUSIONS

Infertility is a global health issue that requires appropriate diagnosis and treatment. Tubal factor being the most common contributing factor in 62% of infertile females in the study. Laparoscopy with chromopertubation and evaluation of associated pelvic pathology remains the gold standard for detection and management of infertility.
